# Interpreting CNN models for musical instrument recognition using multi-spectrogram heatmap analysis: a preliminary study

**DOI:** 10.3389/frai.2024.1499913

**Published:** 2024-12-18

**Authors:** Rujia Chen, Akbar Ghobakhlou, Ajit Narayanan

**Affiliations:** Computer Science and Software Engineering Department, Auckland University of Technology, Auckland, New Zealand

**Keywords:** musical instrument recognition, music information retrieval, convolutional neural networks, spectrogram analysis, feature maps, heatmaps, pattern recognition, feature extraction

## Abstract

**Introduction:**

Musical instrument recognition is a critical component of music information retrieval (MIR), aimed at identifying and classifying instruments from audio recordings. This task poses significant challenges due to the complexity and variability of musical signals.

**Methods:**

In this study, we employed convolutional neural networks (CNNs) to analyze the contributions of various spectrogram representations—STFT, Log-Mel, MFCC, Chroma, Spectral Contrast, and Tonnetz—to the classification of ten different musical instruments. The NSynth database was used for training and evaluation. Visual heatmap analysis and statistical metrics, including Difference Mean, KL Divergence, JS Divergence, and Earth Mover’s Distance, were utilized to assess feature importance and model interpretability.

**Results:**

Our findings highlight the strengths and limitations of each spectrogram type in capturing distinctive features of different instruments. MFCC and Log-Mel spectrograms demonstrated superior performance across most instruments, while others provided insights into specific characteristics.

**Discussion:**

This analysis provides some insights into optimizing spectrogram-based approaches for musical instrument recognition, offering guidance for future model development and improving interpretability through statistical and visual analyses.

## 1 Introduction

Musical instrument recognition is a fundamental aspect of music information retrieval (MIR) research (Downie, 2003; Schedl et al., 2014), aimed at identifying and classifying musical instruments from audio recordings. This task is inherently challenging due to the complexity and variability of musical signals. Different instruments produce unique spectral patterns, which necessitates sophisticated models to accurately recognize them.

Several studies have addressed this challenge by employing convolutional neural networks (CNNs) (LeCun et al., 1989) and different spectrogram representations on various datasets. For instance, some research has classified the open-mic dataset (Humphrey et al., 2018), achieving notable mean per-class accuracy (Chong et al., 2023; Koutini et al., 2021; Schmid et al., 2024; Watcharasupat et al., 2020). Others (Schlüter and Gutenbrunner, 2022; Zeghidour et al., 2021) have evaluated the NSynth (Engel et al., 2017) dataset, while also many works (Avramidis et al., 2021; Kim et al., 2018; Kratimenos et al., 2021; Racharla et al., 2020; Yu et al., 2020) have concentrated on the IRMAS dataset (Bosch et al., 2018).

However, there is a noticeable gap in research specifically examining how different spectrogram representations impact the decision-making process of convolutional neural networks (CNNs) in musical instrument recognition.

### 1.1 Original contribution

In this paper, we explore the use of heatmaps (Chattopadhay et al., 2018; Pan et al., 2021; Qi et al., 2019; Sattarzadeh et al., 2021) to analyse feature contributions across various spectrogram types and instruments also visualize the feature maps (LeCun et al., 2015; Zeiler and Fergus, 2014) to find the different features extracted by CNN kernel.

This study does not propose a new architecture or aim to improve classification accuracy. Instead, it offers a statistical approach for assessing the effectiveness of different spectrogram representations in CNN-based musical instrument recognition. Using metrics such as Difference Mean, Kullback–Leibler Divergence, Jensen-Shannon Divergence, and Earth Mover’s Distance, we quantitatively evaluate feature importance distributions across spectrogram types (e.g., STFT, log-Mel, MFCC). This analysis clarifies how each representation affects classification.

Our findings suggest potential for a label-free loss function based on feature map statistics, which could transition instrument classification from supervised to unsupervised learning by focusing on statistical patterns in feature maps rather than labeled data.

### 1.2 Literature review

CNNs have been extensively employed for various audio recognition tasks due to their ability to capture local patterns in data, which is particularly useful for spectrogram representations of audio signals (Purwins et al., 2019). Early work by Lee et al. (2009) demonstrated the effectiveness of unsupervised feature learning using CNNs for audio classification tasks. They showed that CNNs could learn hierarchical feature representations from raw audio data, leading to improved classification performance. Subsequently, Dieleman and Schrauwen (2014) explored end-to-end learning for music audio using CNNs, highlighting the potential of deep architectures in capturing complex audio features without the need for handcrafted features.

The use of spectrograms as input to CNNs has become a standard approach in audio classification. Spectrograms transform audio signals into a two-dimensional time-frequency representation, making them suitable for CNNs originally designed for image data (Choi et al., 2017). Different types of spectrograms, such as Short-Time Fourier Transform (STFT), Mel-Frequency Cepstral Coefficients (MFCCs), and log-Mel spectrograms, have been investigated to determine their effectiveness in various tasks.

For instance, Sigtia et al. (2016) utilized log-Mel spectrograms for piano transcription, demonstrating improved performance over traditional methods. Similarly, Pons and Serra (2017) compared various spectrogram representations and found that different time-frequency resolutions could capture different musical properties, influencing the classification outcomes.

In the domain of musical instrument recognition, Han et al. (2016) employed CNNs with MFCCs and demonstrated significant improvements in instrument classification accuracy. Their work emphasized the importance of feature representation in conjunction with CNN architecture. Additionally, Gururani et al. (2019) explored the use of transfer learning with pre-trained CNNs on spectrograms for instrument recognition, highlighting the benefits of leveraging models trained on large datasets.

Heatmap analysis methods, such as Class Activation Mapping (CAM) and Integrated Gradients, have been used to interpret CNN models in audio classification tasks (Simonyan and Zisserman, 2014; Sundararajan et al., 2017) These methods provide insights into which parts of the spectrogram contribute most to the classification decisions, aiding in model interpretability.

### 1.3 Convolutional neural networks

Convolutional Neural Networks (CNNs) are neural networks tailored for processing data with a grid-like topology, such as images (LeCun et al., 1998). They use convolutional layers equipped with learnable filters that traverse the input dimensions to generate feature maps, capturing local spatial patterns and preserving spatial hierarchies (Goodfellow et al., 2016). This mechanism enables the detection of features like edges and textures.

Pooling layers follow to reduce the spatial dimensions of feature maps, which lowers computational demands and helps prevent overfitting (Scherer et al., 2010). Common pooling techniques include max pooling and average pooling, which condense information by summarizing regions of the feature maps.

Activation functions such as the Rectified Linear Unit (ReLU) introduce non-linearity into the network, allowing it to model complex patterns (Nair and Hinton, 2010). Fully connected layers at the network’s end aggregate the extracted features to perform tasks like classification.

CNNs have achieved significant success across various fields by effectively learning hierarchical feature representations, establishing themselves as foundational models in deep learning (Krizhevsky et al., 2012).

### 1.4 Spectrogram representations in musical instrument classification

Short-Time Fourier Transform (STFT) provides a detailed time-frequency representation, which has been utilized in several works for musical instrument recognition (Joder et al., 2009; Kim et al., 2018). The logarithmic Mel-frequency (Log-Mel) spectrogram emphasizes perceptually relevant frequency bands, making it a popular choice in instrument classification studies (Castel-Branco et al., 2020, 2021).

Mel-Frequency Cepstral Coefficients (MFCC) efficiently capture the spectral envelope, which has led to its extensive use in musical instrument classification research (Bhalke et al., 2016; Jadhav, 2015; Nagawade and Ratnaparkhe, 2017; Wu et al., 2017). Chroma features, which represent pitch class information, along with Spectral Contrast, which highlights the differences between peaks and valleys in the spectrum, and Tonnetz, which represents harmonic relationships in a pitch space, have also been widely used in music information retrieval (Ezzaidi et al., 2012; Ghosal et al., 2019; Hall et al., 2014; Hook, 2020; Humphrey et al., 2012; Jiang et al., 2002; Karystinaios et al., 2021; Weiß and Habryka, 2014).

### 1.5 Convolutional neural network feature maps and heatmaps of spectrogram image

**Figure 1:**
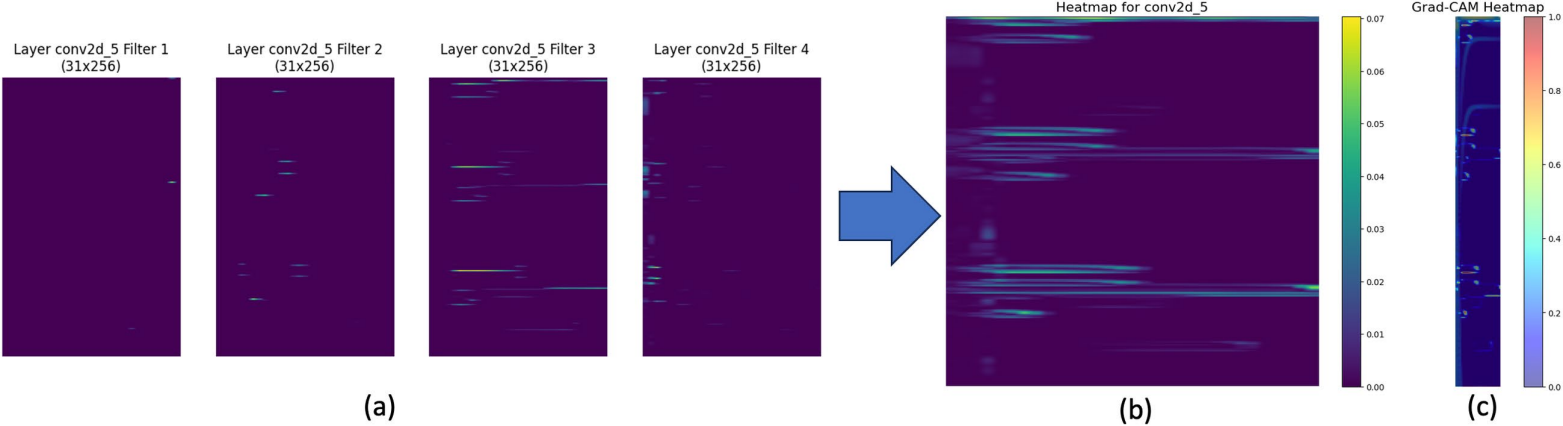
STFT’s Feature Map and Heatmap Analysis for Vocal Instrument. **(a)** Displays four feature maps of layer conv2d_5 filters (filter 1 to filter 4) with dimensions 31x256. **(b)** Shows the corresponding heatmap generated for the conv2d_5 layer. **(c)** Presents the Grad-CAM heatmap, illustrating the model’s focus for classification.

Figure 1 demonstrates the progression from feature maps to heatmaps for vocal instrument classification using STFT and CNN. Panel (a) shows the output of convolutional layers capturing vocal spectrogram patterns. The Integrated Gradients heatmap in panel (b) identifies key time-frequency bins for model prediction. Panel (c) highlights the spectrogram regions crucial for the CNN’s decision, providing insight into the model’s focus during classification.

**Figure 2:**
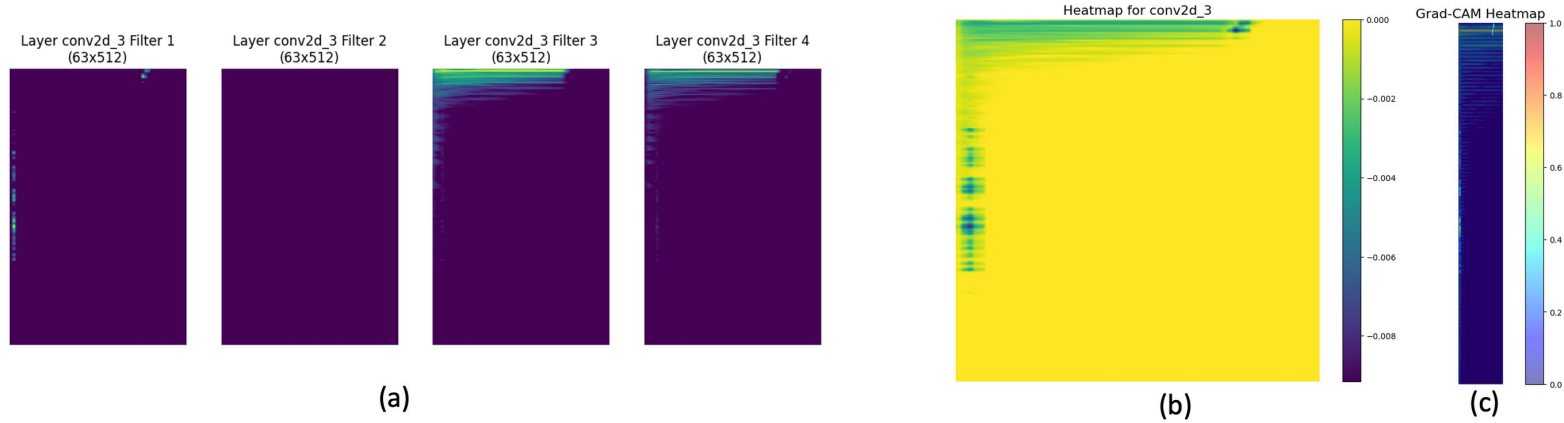
STFT’s Feature Map and Heatmap Analysis for Bass Instrument. **(a)** Displays four feature maps of layer conv2d_3 filters (filter 1 to filter 4) with dimensions 63x512. **(b)** Shows the corresponding heatmap generated for the conv2d_3 layer. **(c)** Presents the Grad-CAM heatmap, highlighting regions of interest for classification.

Figure 2 provides an analysis of feature maps and heatmaps for bass instrument classification using STFT and CNN. Panel (a) illustrates feature maps capturing bass spectrogram patterns. The Integrated Gradients heatmap in panel (b) identifies important time-frequency bins. Panel (c) highlights the spectrogram regions significant for the CNN’s classification decision.

Heatmap analysis is essential for understanding CNN behavior and attention shifts in musical instrument classification. Comparing Figures 1, 2, the attention and feature importance differ between vocal and bass instruments. The vocal instrument (Figure 1) shows a broader spread of significant regions, while the bass instrument (Figure 2) highlights more localized regions. This difference indicates that CNN uses different spectrogram features for each instrument, emphasizing the need for heatmap analysis to interpret and improve model performance accurately. This visual analysis needs to be supplemented with quantitative research to deeply understand the variations in feature importance and model behavior across different instruments. As suggested by Krakov and Feitelson (2013), heatmap comparison can be converted to distribution comparison using algorithms such as Kullback–Leibler (KL) divergence and Jensen-Shannon divergence to provide a more rigorous statistical analysis, enhancing the interpretability and robustness of the model.

## 2 Heatmap evaluation metrics

Despite the advancements of heatmap visualization, there remains a gap in systematically comparing different spectrogram representations and analyzing their impact on CNN-based musical instrument recognition. Existing studies often focus on specific representations or do not delve deeply into the interpretability of the models. This study aims to fill this gap by comprehensively evaluating multiple spectrogram representations (STFT, log-Mel, MFCC) using CNNs and employing advanced heatmap analysis methods to understand feature importance.

**Figure 3:**
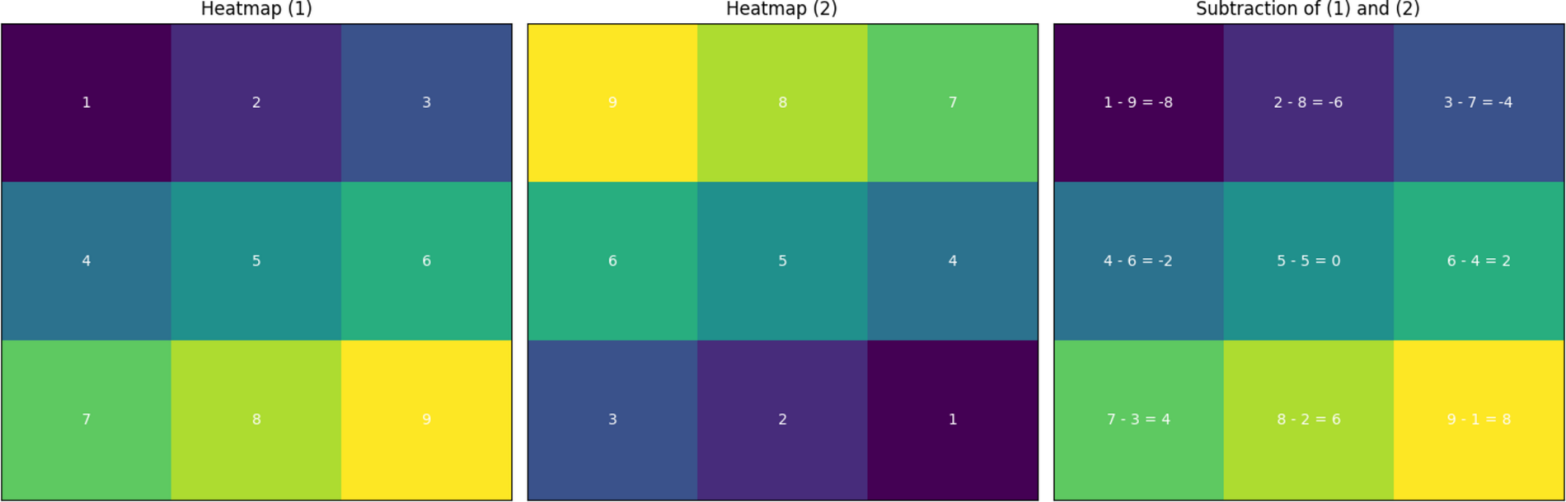
The illustrations of the subtraction process between two heatmaps to analyze differences in feature importance across different conditions. Heatmap (1) and Heatmap (2) show the feature importance distribution for two different spectrogram representations or conditions. The resulting heatmap shows the difference between these two distributions, with each cell annotated to display the specific subtraction calculation (e.g., a1 - b1 = −8).

Figure 3 demonstrates the process of subtracting two heatmaps to highlight differences in feature importance for different spectrogram representations or conditions. In this figure, Heatmap (1) and Heatmap (2) are compared, and the subtraction results are shown in the third panel. Each cell in the subtraction heatmap is annotated with its corresponding calculation (e.g., a1 - b1 = −8), providing a clear understanding of how the subtraction was performed.

This detailed visualization helps in identifying specific areas where feature importance diverges, offering insights into the influence of different spectrogram representations on the model’s decision-making process. To further quantify these differences, additional algorithms such as Kullback–Leibler (KL) divergence and Jensen-Shannon divergence can be employed. These metrics would provide a more rigorous statistical analysis, measuring the similarity and divergence between the heatmaps’ feature importance distributions, thus enhancing the interpretability and robustness of the model.

### 2.1 Difference mean

Difference Mean measures the average difference between two heatmaps. This metric indicates the overall similarity or disparity between the contributions of features in different conditions, such as between different instruments or spectrogram types. A smaller Difference Mean suggests higher consistency in feature importance, while a larger value indicates greater variability.

### 2.2 Kullback–Leibler divergence

KL Divergence (Kullback and Leibler, 1951) quantifies the difference between two probability distributions. When heatmap values are normalized to sum to one, they can be interpreted as probability distributions. KL Divergence measures how one distribution (heatmap) diverges from another, highlighting significant changes in feature contributions. This metric is particularly useful for understanding shifts in model focus under different conditions.

### 2.3 Jensen-Shannon divergence

JS Divergence (Lin, 1991) is a symmetric measure of similarity between two distributions. It provides a more interpretable and stable measure compared to KL Divergence. JS Divergence indicates how similar or different two heatmaps (distributions of feature importance) are, offering insights into the model’s consistency across samples.

### 2.4 Earth Mover’s distance

EMD (Rubner et al., 2000) measures the cost of transforming one distribution into another, considering both the magnitude and distance of differences. This metric offers an intuitive way to quantify similarity by comparing the overall “shape” of feature importance distributions. EMD is useful for assessing the consistency of feature importance across different samples or conditions.

### 2.5 Visual and statistical insights

Heatmaps allow us to visualize which parts of the input, such as specific time-frequency bins in a spectrogram, contribute most to the model’s predictions. This visualization helps us understand the model’s focus and decision-making process. By computing the four metrics between heatmaps for different instruments or spectrogram types, we can quantify how these contributions change. These metrics provide insights into:

- How different spectrogram features contribute to classification accuracy.
- How the model’s attention shifts across different conditions.
- Identifying patterns or anomalies in model behavior.

## 3 Methodology

### 3.1 Data preparation

We utilized a diverse dataset of musical instruments from NSynth Dataset (Engel et al., 2017), converting audio samples into six spectrogram types: Short-Time Fourier Transform (STFT), Log-Mel spectrograms, Mel-Frequency Cepstral Coefficients (MFCCs), Chroma, Spectral Contrast, and Tonnetz. These spectrograms serve as input features for the CNN model, each chosen for its unique representation of audio characteristics, thereby allowing a more comprehensive analysis of CNN interpretability across varied spectrogram inputs.

### 3.2 Convolutional neural network configuration

In our preliminary study on heatmap analysis, we implemented a convolutional neural network with a total of 10 layers, including 6 convolutional layers for feature extraction and 4 additional layers for pooling, dropout, and dense processing. The network begins with an input layer tailored to the heatmap input shape, followed by three blocks of convolutional layers: each block contains two convolutional layers (using 32, 64, and 128 filters respectively) with a kernel size of 3×3, ReLU activation, and same padding. Max pooling layers, each with a 2×2 pool size, follow each block to reduce spatial dimensions, and dropout layers with rates of 0.25 after each block help prevent overfitting.

The flattened output from the convolutional blocks is fed into a fully connected layer with 256 neurons and a dropout rate of 0.5 to further enhance generalization. Finally, a dense layer with 1 neuron and sigmoid activation provides binary classification. The model is compiled with the Adam optimizer, binary cross-entropy loss, and an accuracy metric to assess interpretability and classification performance on the heatmap data.

### 3.3 Integrated gradients heatmap

To gain insights into the CNN’s decision-making process, we generated heatmaps for each spectrogram type using the Integrated Gradients method. This technique attributes the CNN’s predictions to its input features by computing gradients along the path from a baseline input to the actual input. The resulting heatmaps identify which parts of the spectrogram contribute most to model predictions, facilitating a deeper understanding of model interpretability. We selected Integrated Gradients for its ability to provide consistent attributions across different spectrogram types, making it suitable for evaluating feature importance distribution across audio representations.

### 3.4 Experiment design

The experimental framework for training and testing the CNN models on each spectrogram type is detailed in Algorithm 1. For each spectrogram type (STFT, Log-Mel, MFCC, Chroma, Spectral Contrast, and Tonnetz), CNN models were trained and validated across a range of instruments, including Bass, Brass, Flute, Guitar, Keyboard, Mallet, Organ, Reed, String, and Vocal. Each model underwent training for 200 epochs with a 20% validation split, ensuring sufficient learning and performance assessment across instruments. Post-training, models were tested on separate test sets, and accuracy metrics were recorded. This process aims to confirm the robustness of the models in recognizing musical instruments based on varied spectrogram features.

#### Algorithm 1 Training and testing CNN models

**Table 1:** 

1: **for** each type *t* in (STFT, Log-Mel, MFCC, Chroma, Spectral Contrast, Tonnetz) **do** 2:**for** each instrument *i* in (Bass, Brass, Flute, Guitar, Keyboard, Mallet, Organ, Reed, String, Vocal) **do** 3:**Split** dataset for instrument *i* into training and validation sets (validation_split = 0.2) 4:**Train and validate** the *CNN model* for instrument *i* with spectrogram type *t* using the training and validation sets for 200 epochs 5:**Test** *CNN model* for instrument *i* with spectrogram type *t* using the test set 6:**Calculate** and record accuracy for instrument *i* with spectrogram type *t* 7:**end for** 8: **end for**

(*Algorithm 1 outlines the process for training and validating CNN models across various spectrogram types and instruments*.)

To systematically evaluate the feature importance highlighted by the CNN’s heatmaps, we applied four statistical metrics: Difference Mean, Kullback–Leibler (KL) Divergence, Jensen-Shannon (JS) Divergence, and Earth Mover’s Distance (EMD). These metrics quantify the consistency and variability of feature importance distributions across different samples, offering a nuanced understanding of how the model interprets each spectrogram type for instrument classification.

Algorithm 2 illustrates the statistical analysis process. For each instrument, corresponding heatmaps were analyzed by calculating the Difference Mean, KL Divergence, JS Divergence, and EMD for each sample pair. This analysis provides a quantitative assessment of feature stability and distribution shifts, which are essential for improving model interpretability. The use of these metrics allows us to evaluate the model’s focus areas more rigorously, identify patterns, and uncover limitations in spectrogram representation, informing potential improvements.

#### Algorithm 2 Heatmap statistical analysis

**Table 2:** 

1: **for** each instrument *i* in instruments **do** 2:**Load** heatmaps for instrument *i* 3:**for** each sample *s₁* in heatmaps **do** 4:**for** each sample *s₂* in heatmaps where *s₂* ≠ *s₁* **do** 5:**Calculate** *difference mean* between *s₁* and *s₂* 6:**Calculate** *KL divergence* between *s₁* and *s₂* 7:**Calculate** *Jensen-Shannon divergence* between *s₁* and *s₂* 8:**Calculate** *Earth Mover’s Distance* between *s₁* and *s₂* 9:**end for** 10:**end for** 11: **end for**

(*Algorithm 2 outlines the statistical analysis steps used for comparing heatmap samples across different instruments, calculating various divergence metrics and distances to evaluate similarity*.)

Through these steps, our methodology balances rigorous CNN training with in-depth heatmap analysis to offer initial insights into feature importance distributions across multiple spectrogram representations. While we acknowledge the basic nature of these methods in this preliminary study, we recognize the potential for integrating more sophisticated techniques in future work to enhance model interpretation further.

### 3.5 Extraction and analysis of heatmap interpretability using integrated gradients

The heatmaps were extracted using the Integrated Gradients method, implemented with Python and TensorFlow. Integrated Gradients is a technique for interpreting neural network predictions by quantifying the contribution of each input feature to the final prediction. For each input sample, we generated a heatmap by interpolating between a baseline input (a zero-valued matrix) and the actual input, creating a series of scaled inputs that gradually shift from the baseline to the original input.

The gradient calculation for each scaled input was performed using TensorFlow’s GradientTape context, capturing and computing the gradients of the model’s prediction with respect to each interpolated input. We then averaged these gradients and multiplied them by the difference between the actual input and the baseline to produce the final integrated gradient heatmap.

## 4 Result

### 4.1 Classification result per spectrogram

**Figure 4:**
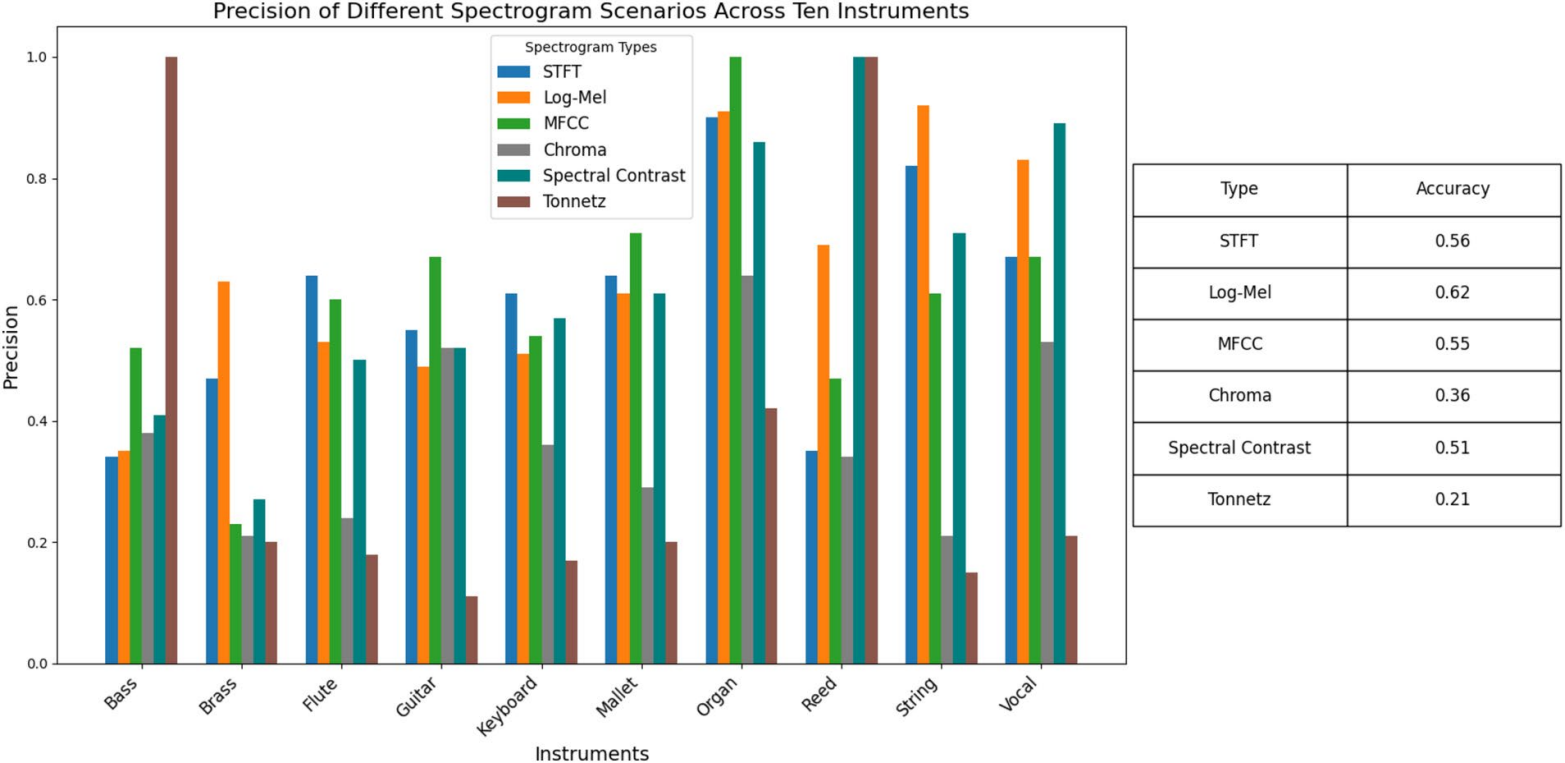
Precision of different spectrogram scenarios across 10 instruments. This bar chart illustrates the precision of CNN models trained on six spectrogram types for ten different musical instruments.

Figure 4 displays the classification results of CNN models trained on six different spectrogram types across 10 musical instruments. The precision values indicate the proportion of true positive predictions among all positive predictions made by the models. From the bar chart, it is evident that certain spectrogram types perform better for specific instruments. For instance, the Tonnetz spectrogram exhibits high precision for Brass and Reed instruments, while the STFT spectrogram shows lower precision for these instruments. In contrast, the MFCC spectrogram demonstrates consistently high precision for most instruments, particularly for Mallet and Organ. This visual comparison helps in identifying the most effective spectrogram type for each instrument, providing valuable insights for optimizing musical instrument recognition models.

The MFCC spectrogram achieves the highest overall accuracy (0.62), indicating its robustness across various instruments. The Log-Mel spectrogram also performs well, with an overall accuracy of 0.55, particularly excelling in Flute and Mallet instrument classification. The STFT spectrogram, while showing decent performance for some instruments like Organ and Reed, has a lower overall accuracy (0.56). The Chroma spectrogram exhibits moderate performance, with significant variability across instruments, achieving the highest precision for Brass but lower values for others like Vocal and String. The Spectral Contrast and Tonnetz spectrograms show variable performance, with Tonnetz achieving high precision for Reed but low values for other instruments. These metrics suggest that while MFCC and Log-Mel spectrograms are generally effective for musical instrument recognition, the choice of spectrogram type can significantly impact model performance depending on the specific instrument being classified. This analysis underscores the importance of selecting appropriate spectrogram representations to enhance the accuracy and reliability of musical instrument recognition models.

Leaf (Zeghidour et al., 2021) achieved 69.2% accuracy, while Efficient Leaf (Schlüter and Gutenbrunner, 2022) reached 71.7% accuracy in their respective benchmarks. However, this paper does not aim to surpass existing benchmark results, but rather focuses on interpreting CNN-generated heatmaps through the analysis of four key metrics: Difference Mean, KL Divergence, JS Divergence, and Earth Mover’s Distance. Our primary goal is to provide insights into the model’s decision-making process by evaluating the feature importance of different spectrogram types. By concentrating on interpretability rather than performance benchmarking, this study contributes to a deeper understanding of how CNNs utilize spectrogram features for musical instrument recognition.

### 4.2 Heatmap analysis result

#### 4.2.1 Short-time fourier transform heatmap analysis based on four metrics

Difference Mean of Bass and brass instruments show the smallest value, indicating high consistency in feature importance across samples. Vocal and string instruments have the largest difference means, suggesting more variability in how the model identifies these instruments. Mallet and keyboard instruments show moderate variability. Bass shows high KL divergence, indicating that despite low mean differences, the distribution of important features varies significantly between samples. Organ and reed instruments have lower KL divergence, suggesting more consistent feature importance distributions. Guitar and flute show moderate KL divergence. JS Divergence of most instruments cluster in the 0.4–0.6 range, with bass showing slightly higher divergence. Organ has the lowest JS divergence, indicating more consistent feature importance across samples.

Bass shows the highest EMD, suggesting that the important features for classification are more spread out or shifted between samples. Vocal and organ show lower EMD, indicating more localized and consistent important features.

**Figure 5:**
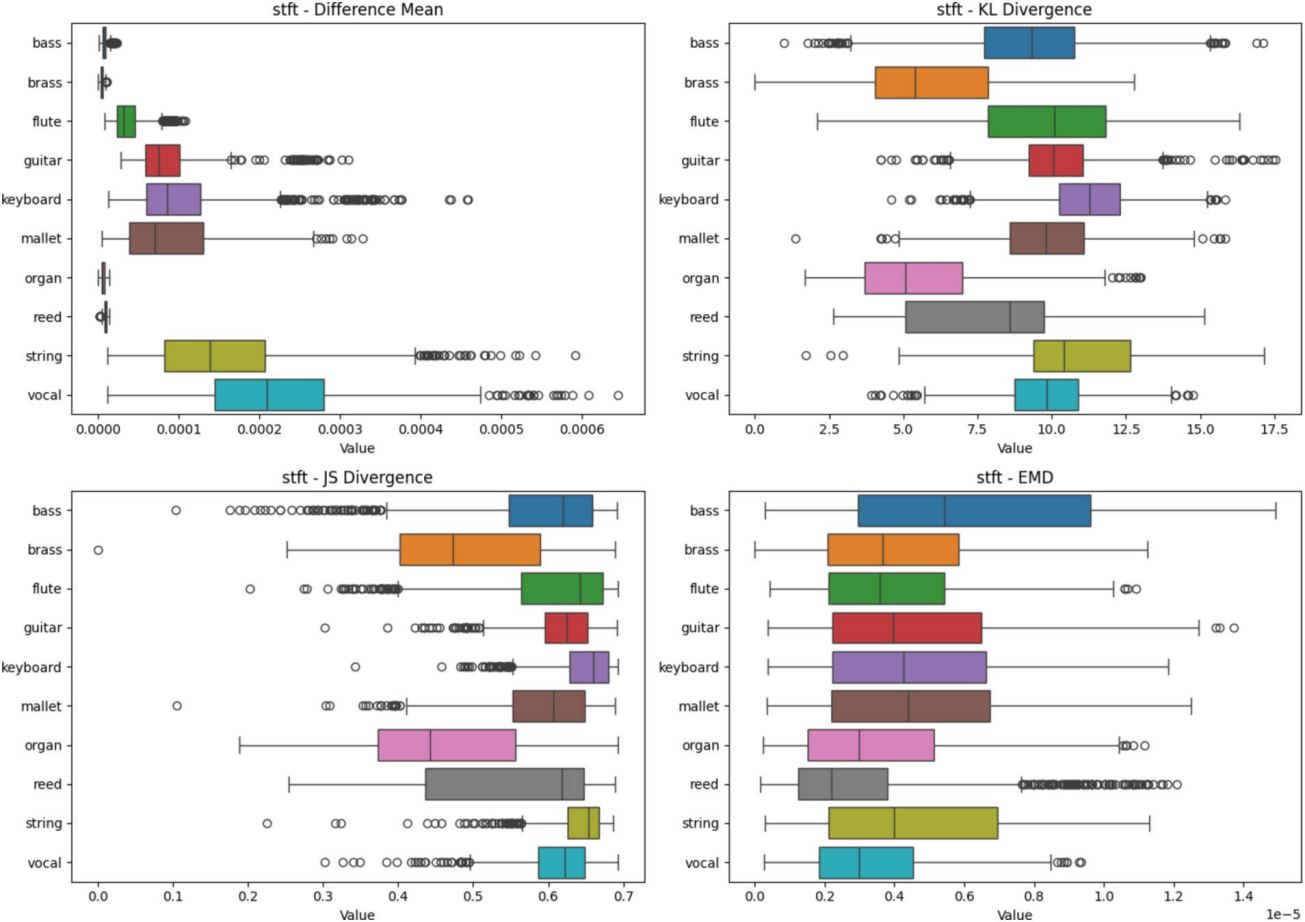
Heatmap analysis of different spectrogram scenarios across 10 instruments. These boxplots illustrate the statistical metrics (Difference Mean, KL Divergence, JS Divergence, and EMD) for the feature importance heatmaps generated from CNN models using STFT Spectrogram.

Instruments like organ and reed seem to have more consistent feature importance across samples, making them potentially easier for the model to classify reliably. Bass, despite low mean differences, shows high variability in feature distribution, which might explain challenges in its classification. The high EMD for bass suggests that important features for its classification are more dispersed or variable in the spectrogram, potentially making it harder for the model to learn robust features. Vocal and string instruments show higher variability across metrics, suggesting that their classification relies on a more diverse set of features that may vary between samples. These insights can guide improvements in the model or feature extraction process (Figure 5).

#### 4.2.2 Log-mel heatmap analysis based on four metrics

String instruments show the highest difference mean, indicating significant variability in feature importance across samples. Bass, brass, flute, and guitar have the lowest difference means, suggesting more consistent feature importance. Organ and mallet instruments show moderate variability. Bass and flute exhibit high KL divergence, indicating that despite low mean differences, the distribution of important features varies significantly between samples. Organ and string instruments show lower KL divergence, suggesting more consistent feature importance distributions. Guitar and keyboard have moderate KL divergence. Most instruments cluster in the 0.5–0.6 range. Bass shows the highest JS divergence, indicating less consistency in feature importance across samples. String instruments have the lowest JS divergence, suggesting more consistent feature importance patterns. Bass shows the highest EMD, suggesting that important features for classification are more spread out or shifted between samples. Flute and reed show lower EMD, indicating more localized and consistent important features. Guitar, keyboard, and mallet instruments have moderate EMD values.

**Figure 6:**
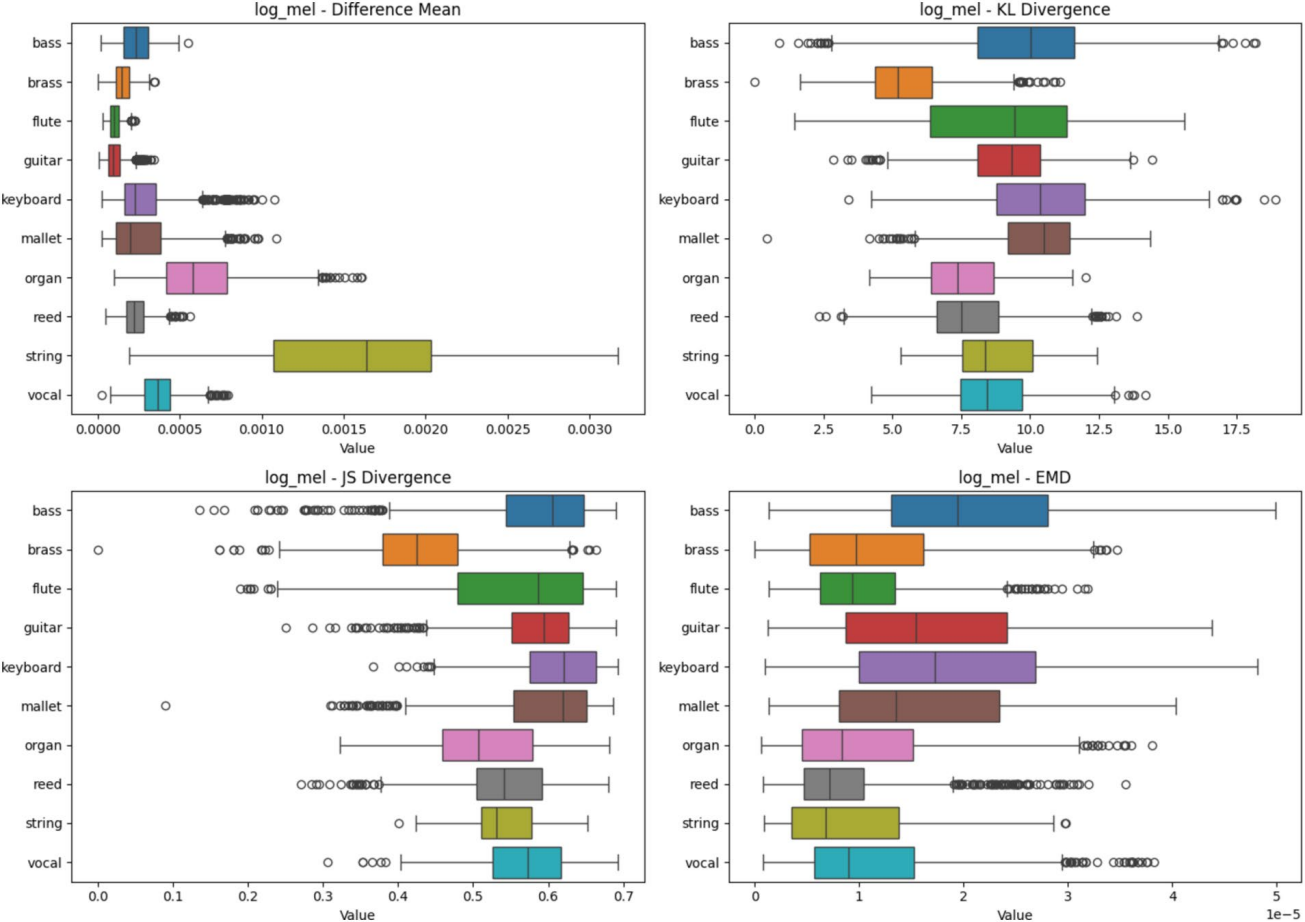
Heatmap Analysis of different spectrogram scenarios across 10 instruments. These boxplots illustrate the statistical metrics (Difference Mean, KL Divergence, JS Divergence, and EMD) for the feature importance heatmaps generated from CNN models using Log-Mel Spectrogram.

Instruments like flute and reed demonstrate more consistent feature importance across samples in the log-mel domain, potentially leading to more reliable classification. Bass exhibits high variability across all metrics, suggesting that its spectral characteristics in the log-mel domain are less consistent or more complex, which may challenge classification. String instruments show high difference mean but lower divergence measures, indicating that while the magnitude of important features varies, their distribution remains relatively consistent. This could reflect the complex harmonic structure of string instruments captured by log-mel spectrograms. The log-mel spectrogram’s non-linear frequency resolution might be particularly beneficial for instruments like organ and keyboard, as evidenced by their moderate to low variability across metrics (Figure 6).

#### 4.2.3 Mel-frequency cepstral coefficient heatmap analysis based on four metrics

**Figure 7:**
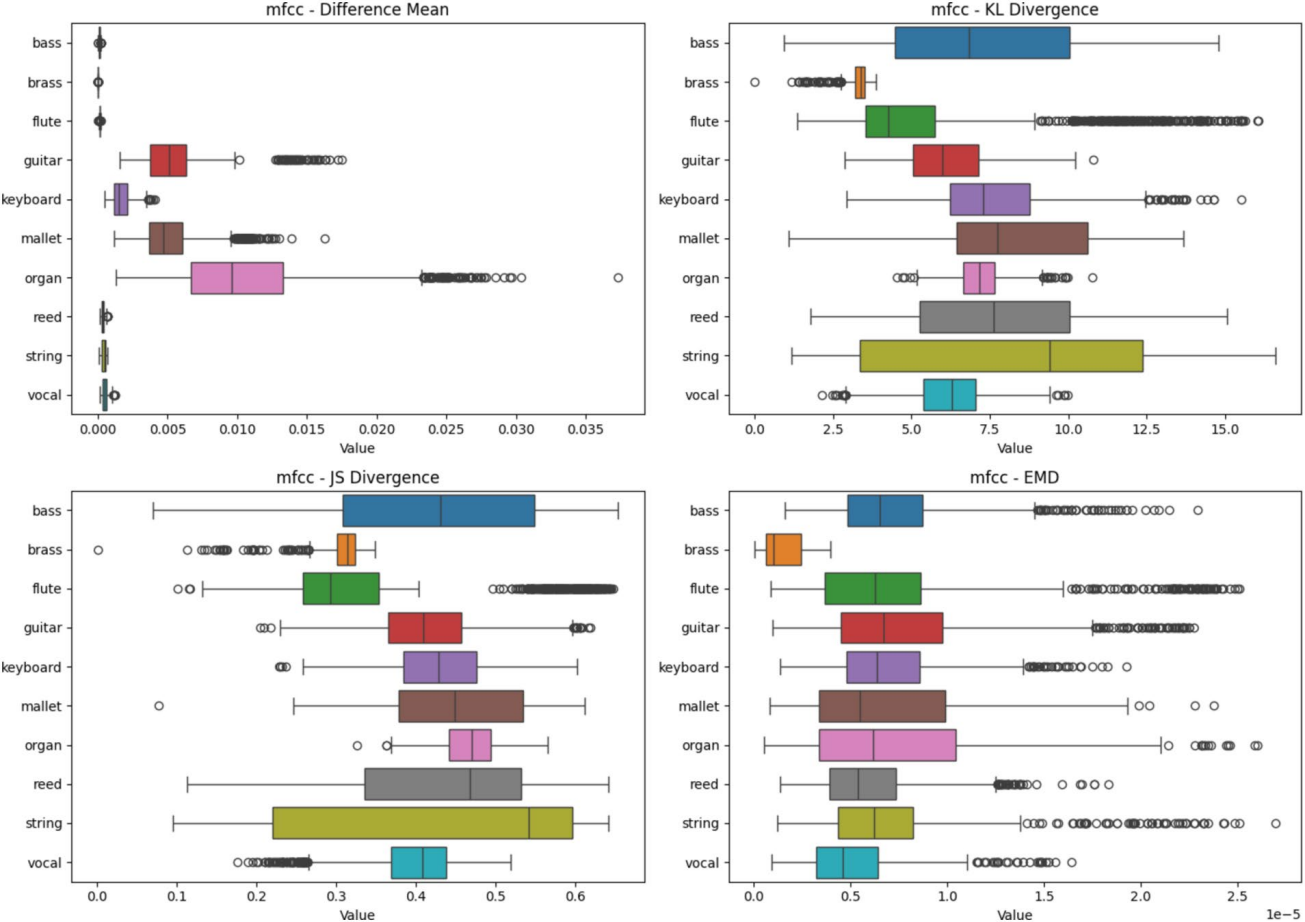
Heatmap analysis of different spectrogram scenarios across 10 instruments. These boxplots illustrate the statistical metrics (Difference Mean, KL Divergence, JS Divergence, and EMD) for the feature importance heatmaps generated from CNN models using MFCC Spectrogram.

Figure 7 keyboard shows the highest difference mean with a widespread, indicating significant variability in feature importance across samples. Brass, flute, mallet, reed, and string have very low difference means, suggesting highly consistent feature importance. Bass and guitar show moderate variability. Bass exhibits high KL divergence, indicating that the distribution of important features varies significantly between samples. Organ shows the lowest KL divergence, suggesting more consistent feature importance distributions. Most instruments have relatively high KL divergence values, indicating varied consistency in feature distributions. Most instruments cluster in the 0.5–0.7 range, indicating moderate consistency in feature importance across samples. Reed shows the highest JS divergence, suggesting less consistency in feature importance patterns. Brass has the lowest JS divergence, indicating more consistent feature importance patterns. Bass shows the highest EMD values, suggesting that important features for classification are spread out or shifted between samples. Organ shows the lowest EMD, indicating more localized and consistent important features. String instruments have a wide range of EMD values, suggesting varied feature importance locations.

Brass and flute demonstrate consistent feature importance in the Chroma domain, potentially due to their well-defined pitch characteristics. Keyboard shows high variability across metrics, indicating that its harmonic content in the Chroma domain is complex and varied, which may present challenges for classification. Bass exhibits moderate difference mean but high variability in other metrics, suggesting that while the magnitude of important features is somewhat consistent, their distribution varies significantly. This could reflect the challenge of representing low-frequency content in Chroma features. Guitar and keyboard show more variability, possibly due to their polyphonic nature and the way Chroma features capture harmonic content.

#### 4.2.4 Chroma heatmap analysis based on four metrics

**Figure 8:**
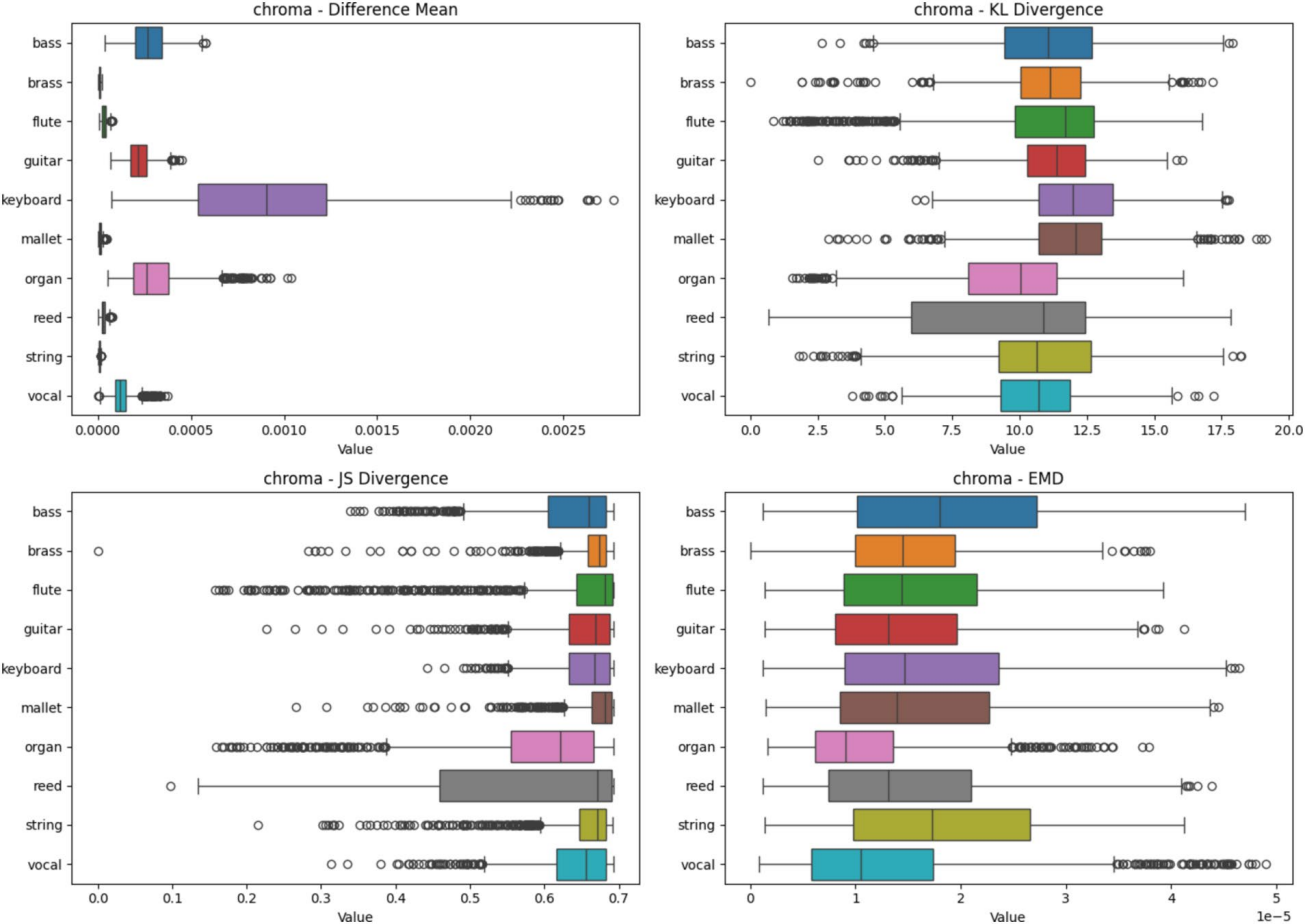
Heatmap analysis of different spectrogram scenarios across 10 instruments. These boxplots illustrate the statistical metrics (Difference Mean, KL Divergence, JS Divergence, and EMD) for the feature importance heatmaps generated from CNN models using Chroma Spectrogram.

Figure 8 presents the heatmap analysis for various instruments using Chroma spectrograms. The boxplots display statistical metrics, including Difference Mean, KL Divergence, JS Divergence, and EMD. These metrics indicate the consistency of feature importance across different samples. Instruments like Brass and Flute exhibit high consistency, while others, like Keyboard, show significant variability, reflecting the harmonic complexity captured by Chroma features.

#### 4.2.5 Spectral contrast heatmap analysis based on four metrics

**Figure 9:**
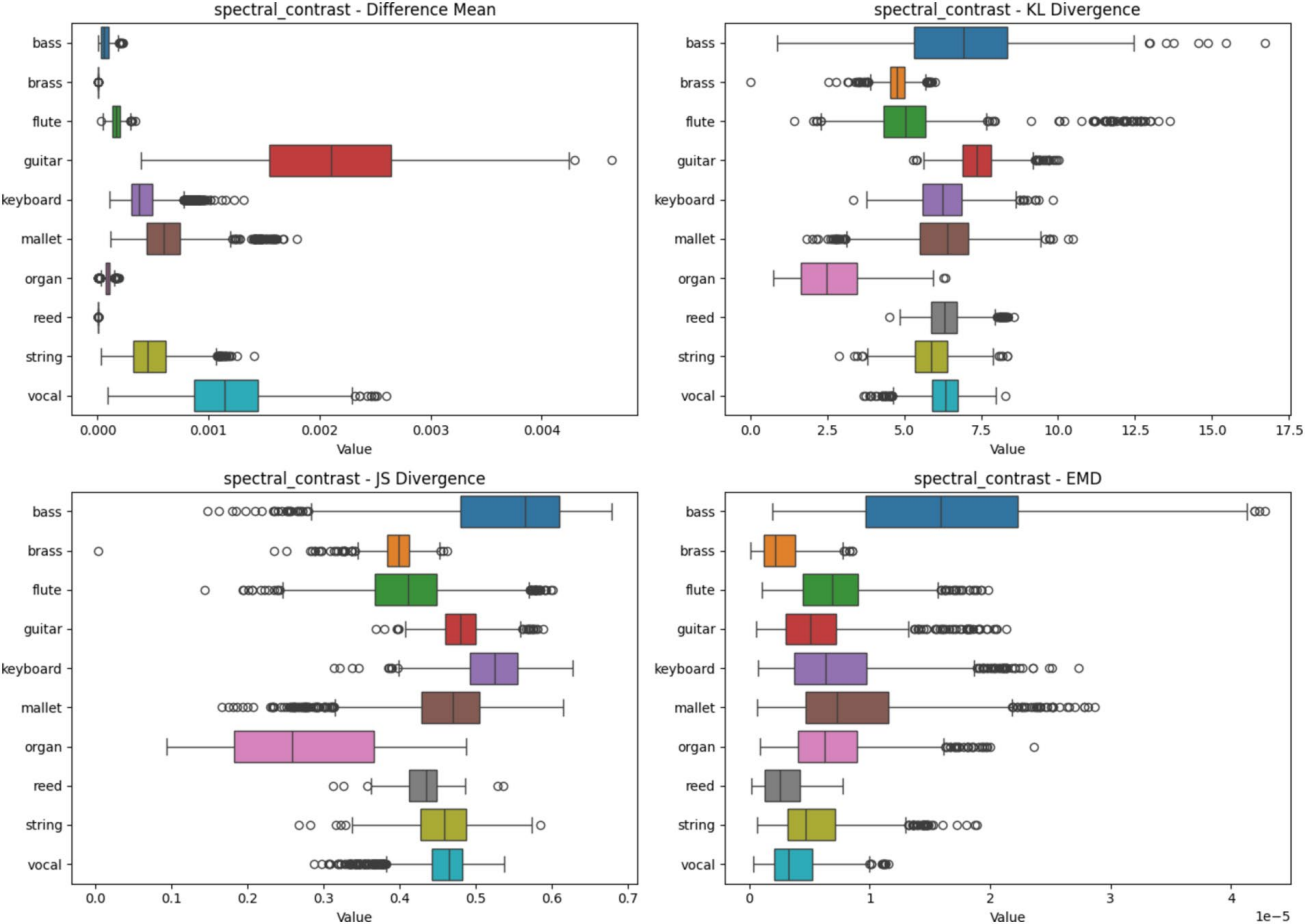
Heatmap analysis of different spectrogram scenarios across 10 instruments. These boxplots illustrate the statistical metrics (Difference Mean, KL Divergence, JS Divergence, and EMD) for the feature importance heatmaps generated from CNN models using Spectral Contrast Spectrogram.

Figure 9 illustrates the heatmap analysis using Spectral Contrast spectrograms. The boxplots highlight the statistical metrics for different instruments, revealing how feature importance varies. Instruments like Reed and Organ show low variability, indicating consistent feature importance across samples, whereas Bass and Guitar display higher variability, suggesting that Spectral Contrast captures distinctive timbral features essential for these instruments’ classification.

#### 4.2.6 Tonnetz heatmap analysis based on four metrics

**Figure 10:**
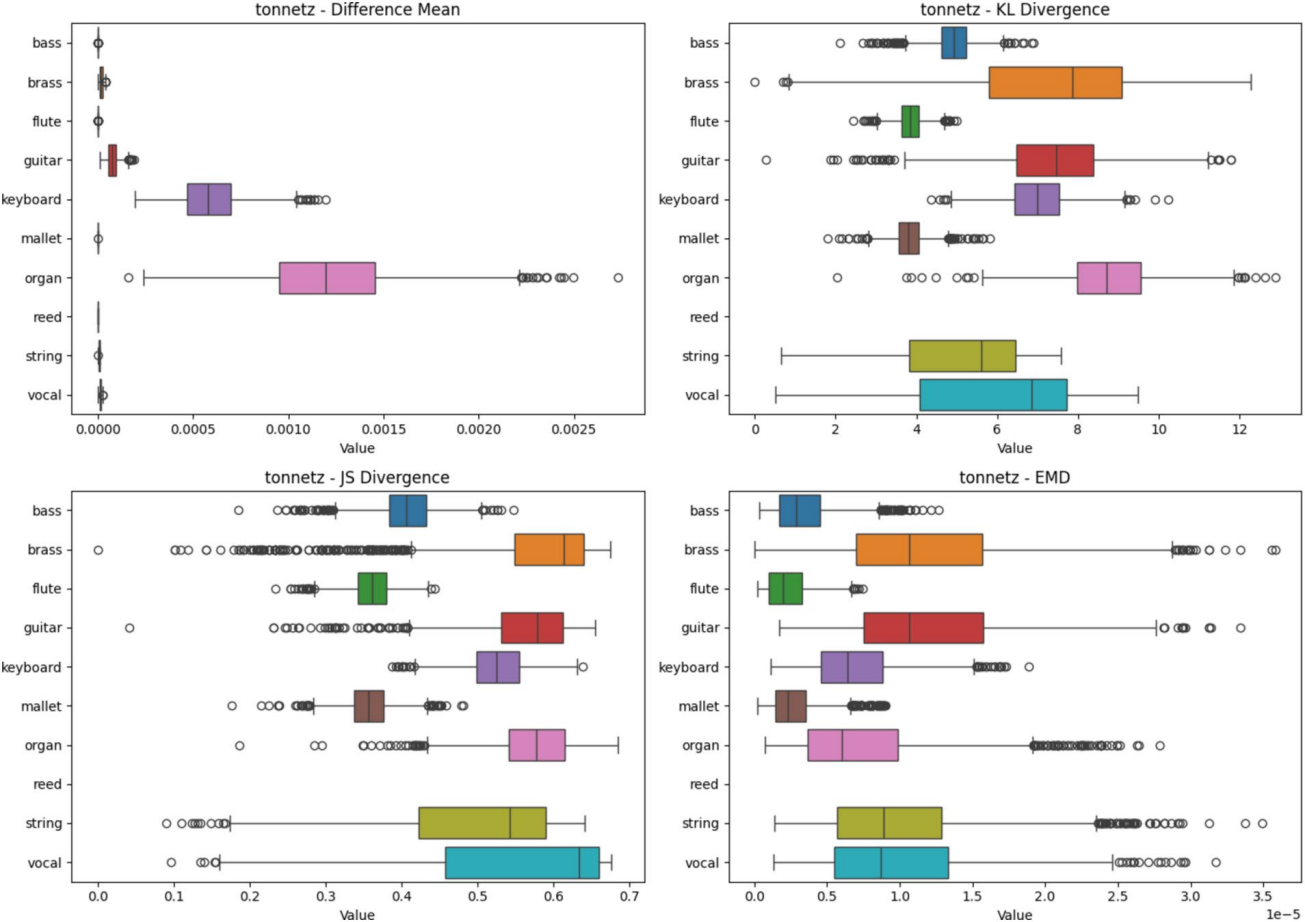
Heatmap analysis of different spectrogram scenarios across 10 instruments. These boxplots illustrate the statistical metrics (Difference Mean, KL Divergence, JS Divergence, and EMD) for the feature importance heatmaps generated from CNN models using Tonnetz Spectrogram.

Figure 10 showcases the heatmap analysis with Tonnetz spectrograms. The boxplots represent statistical metrics for various instruments. Reed and Brass instruments demonstrate high consistency in feature importance, while Bass and Vocal instruments show more variability. This analysis underscores Tonnetz’s effectiveness in capturing harmonic relationships for specific instruments, aiding in their reliable classification.

## 5 Discussion

The analysis reveals that certain spectrogram types are more effective for classifying specific instruments. MFCC spectrograms provide consistently high precision for Bass, Flute, Mallet, Organ, and Vocal instruments, capturing essential spectral features efficiently. However, Bass exhibits significant variability in STFT and Tonnetz spectrograms, indicating that these types are less stable for Bass classification. Brass instruments achieve high precision with Chroma and Tonnetz spectrograms, effectively capturing pitch class and harmonic relationships. Log-Mel spectrograms align well with human auditory perception, making them particularly effective for Flute and Keyboard classification. Despite the complexity of Guitar’s timbral characteristics, MFCC shows some consistency, while other spectrograms result in variable performance.

Reed instruments are best classified using Tonnetz and Spectral Contrast spectrograms, capturing harmonic and timbral features effectively. String instruments show high variability in STFT and Chroma spectrograms but perform moderately with Log-Mel spectrograms, indicating the complexity of their harmonic structure. Mallet instruments achieve high precision with both MFCC and Log-Mel spectrograms, while Organ demonstrates robust classification with MFCC and Log-Mel but variability in STFT. Vocal classification remains challenging across all spectrogram types, though MFCC provides the best performance by capturing the spectral envelope effectively. These findings underscore the importance of selecting appropriate spectrogram types tailored to the specific characteristics of each instrument to optimize classification performance.

### 5.1 Practical implications for MIR systems

The spectrogram effectiveness findings could inform feature extraction in MIR systems. For example, systems focusing on Bass or Flute might prioritize MFCC features, while those dealing with Brass could benefit from Chroma and Tonnetz features. This approach may improve accuracy in instrument recognition tasks.

MIR systems could potentially adapt by selecting or weighting spectrogram representations based on audio input. This might enhance performance across diverse musical content, potentially improving applications like automated track tagging or music recommendation. In addition, the heatmap analysis technique could be integrated into MIR system development, offering a method to interpret CNN behavior. This may help developers identify model biases or weaknesses, potentially leading to more transparent systems.

## 6 Conclusion

Our research provides a comprehensive analysis of feature contributions in musical instrument recognition using different spectrogram types based on NSynth samples. By employing integrated gradients, we generated heatmaps that highlight the parts of the spectrograms contributing most to the model’s predictions. We then quantified the differences between these heatmaps using metrics such as Difference Mean, KL Divergence, JS Divergence, and EMD. These findings can contribute to a deeper understanding of model behavior in musical instrument recognition. By examining how different spectrogram features influence model predictions, we can identify strengths and weaknesses in the feature extraction process. This knowledge can guide improvements in model design and feature engineering, ultimately enhancing the accuracy and reliability of musical instrument recognition systems using real musical samples.

Integrating STFT, log-mel, MFCC, chroma, spectral contrast, and Tonnetz into a single composite image creates a comprehensive multi-spectral representation of the audio signal, enhancing the CNN’s ability to recognize different instruments. This approach allows the network to learn from various aspects of the audio simultaneously, leading to improved accuracy, robustness to variations in audio quality, and effective feature learning. However, this strategy also introduces challenges, such as increased computational complexity, higher risk of overfitting, and difficulties in interpretability. Heatmap analysis must now reflect the importance of features across all combined spectrograms, necessitating the development of methods to visualize and interpret the importance of different spectrogram types within the combined representation.

Thus, future work could explore weighted combinations of spectrogram types, implementing attention mechanisms to focus on the most relevant parts of the combined spectrogram for each instrument, and using a multi-stream architecture where parallel CNN streams for each spectrogram type combine their features later in the network. Additionally, methods to isolate the contribution of each spectrogram type to the final classification decision should be developed. A concatenated CNN with attention layers could further identify which parts of the spectrograms are most influential in the model’s decision-making process, enhancing the interpretability and effectiveness of multi-spectral representation in musical instrument recognition.

## 7 Limitations

This study has certain limitations that may impact on the generalizability and applicability of its findings. First, the dataset used in this research is a subsample of the NSynth dataset, which, though suitable for initial analysis, is relatively small and limited in scope. This restricted sample size limits the extent to which the findings can be generalized across broader contexts.

Additionally, the study relies on synthesized audio samples from the NSynth dataset, which, while normalized for volume consistency and free from spectrogram distortions, does not fully replicate the characteristics of real-world audio recordings. As a result, validation with real audio samples is necessary to confirm the robustness of the proposed methodology. Finally, the current experiment does not consider more complex audio scenarios, such as overlapping instruments or sound variations (e.g., instruments fading in and out), which could provide deeper insights into the model’s adaptability to diverse auditory conditions.

## 8 Future work

Building on the insights gained from this preliminary study, future work will focus on several enhancements to improve the robustness and applicability of the findings. Expanding the dataset to include a larger variety of real-world audio samples will be a primary objective, allowing for better generalizability and validation across different audio conditions. Additionally, incorporating more complex audio scenarios, such as overlapping instruments and varying sound intensities, could offer a more comprehensive assessment of the model’s performance and interpretability under realistic conditions.

Another key direction will be to refine and apply the statistical metrics developed here to evaluate feature importance in diverse datasets, potentially extending to a label-free, heatmap-driven loss function. This approach could reduce reliance on labeled data, moving toward unsupervised or semi-supervised learning frameworks that leverage the statistical patterns identified in heatmaps. These extensions will enhance the study’s contributions to CNN-based musical instrument recognition and aid in developing more interpretable and adaptive models for audio classification.

## Data Availability

The original contributions presented in the study are included in the article/Supplementary material, further inquiries can be directed to the corresponding author/s.
