# Supplementary material for: Interpreting CNN models for musical instrument recognition using multi-spectrogram heatmap analysis: a preliminary study
Source: Front Artif Intell. 2024 Dec 18;7:1499913. doi: 10.3389/frai.2024.1499913 (PMC11688478; doi:10.3389/frai.2024.1499913)
Supplement: Supplementary file 1 [file Table_1.docx]

Supplementary Material

# Supplementary Data

This study utilizes the publicly available NSynth Dataset (Engel et al., 2017) for musical instrument recognition. The dataset can be accessed via the following link:

NSynth Dataset: <https://magenta.tensorflow.org/datasets/nsynth>

In addition to the dataset, all the code used to reproduce the experiments and results from this paper can be found in the following GitHub repository:

GitHub Repository: <https://github.com/fireHedgehog/music-intrument-OvA-model/tree/main/multi-spectrogram-experiment>

# Supplementary Figures and Tables

There are no supplementary figures or tables included in this submission.

3. Reference

Engel, J., Resnick, C., Roberts, A., Dieleman, S., Norouzi, M., Eck, D., & Simonyan, K. (2017). Neural audio synthesis of musical notes with wavenet autoencoders. *International Conference on Machine Learning*, 1068–1077.
